# How cold is it? TRPM8 and TRPA1 in the molecular logic of cold sensation

**DOI:** 10.1186/1744-8069-1-16

**Published:** 2005-04-22

**Authors:** David D McKemy

**Affiliations:** 1 Department of Biological Sciences, Neurobiology Section and School of Dentistry. University of Southern California, 925 West 34^th ^Street, Room 4110, Los Angeles, CA 90089-0641, USA

## Abstract

Recognition of temperature is a critical element of sensory perception and allows us to evaluate both our external and internal environments. In vertebrates, the somatosensory system can discriminate discrete changes in ambient temperature, which activate nerve endings of primary afferent fibers. These thermosensitive nerves can be further segregated into those that detect either innocuous or noxious (painful) temperatures; the latter neurons being nociceptors. We now know that thermosensitive afferents express ion channels of the transient receptor potential (TRP) family that respond at distinct temperature thresholds, thus establishing the molecular basis for thermosensation. Much is known of those channels mediating the perception of noxious heat; however, those proposed to be involved in cool to noxious cold sensation, TRPM8 and TRPA1, have only recently been described. The former channel is a receptor for menthol, and links the sensations provided by this and other cooling compounds to temperature perception. While TRPM8 almost certainly performs a critical role in cold signaling, its part in nociception is still at issue. The latter channel, TRPA1, is activated by the pungent ingredients in mustard and cinnamon, but has also been postulated to mediate our perception of noxious cold temperatures. However, a number of conflicting reports have suggested that the role of this channel in cold sensation needs to be confirmed. Thus, the molecular logic for the perception of cold-evoked pain remains enigmatic. This review is intended to summarize our current understanding of these cold thermoreceptors, as well as address the current controversy regarding TRPA1 and cold signaling.

## Introduction

Our perception of temperature is a finely tuned element of our somatosensory system, fundamentally allowing us to avoid thermal conditions that may be potentially harmful in nature. The preponderance of studies into thermosensation have focused on noxious heat, with the best characterized populations of thermosensitive afferents being those that have 'moderate' and 'high' heat thresholds of ~43 and 52°C, respectively [1]. The first insights into the molecules mediating thermosensation came from the cloning of the capsaicin receptor, or TRPV1, a non-selective cation channel activated by temperatures in the "moderate" heat range [2], and a TRPV1 homologue, TRPV2, that responds to temperatures near the "high" heat thresholds [3]. Taken together, these channels are considered critical for our perception of noxious, painful heat and provided the first clues in the molecular logic for thermosensation [4].

## When do we feel cold?

In contrast to the definitive thermal thresholds of noxious heat-sensitive nerves, similar distinctions for cool- and noxious cold-sensitive fibers have been problematic. In general, the perception of non-painful, cool temperatures is reported to occur when the skin is cooled as little as 1°C from normal body temperature [5]. In fiber recordings, temperatures in the range of 30–15°C will activate both Aδ- and C-fibers [5-7]. These cold-sensitive afferents will fire continuously at body temperature, with cold stimuli inducing an increase in their rate of firing, while warm temperatures reduce this activity [5]. However, peak responsiveness can vary between studies, falling between the temperatures of 25 and 15°C [5,8]. Once temperatures approach 15°C, the perception of cold pain is evoked, with qualities described as burning, aching, and pricking [9]. However, the exact proportion of nociceptors that respond to noxious cold temperatures is not clear, with reported percentages ranging from 10 to 100% of Aδ- and C-fibers [6,10-12]. Thus, it has been difficult to group cold-sensitive afferents in a manner similar to the distinct categorizations made of heat-sensitive fibers.

More recently, a number of laboratories have studied cold using cultured dorsal root (DRG) or trigeminal (TG) ganglion neurons as *in vitro *models of afferent nerves. In contrast to fiber recordings, it is consistently reported that approximately 10–20% of these cells will respond to cold temperatures, with thresholds for activation ranging from ~30 to near 15°C [13-16]. Moreover, two groups of neurons with distinct cold responses have been described, the predominant response characterized by a low-activation threshold temperature near 30°C and a second class of neurons with a high-activation threshold below 20°C [15,17,18]. Thus, the different activation thresholds suggest the former cells to be an *in vitro *model for innocuously cool signaling afferents, while the latter may be analogous to those mediating noxious cold. Moreover, each neuronal population has a distinct response profile that suggests cold elicits these effects through different mechanisms (see below). Thus, these cultured sensory afferents have been a useful experimental model and provided insights into the mechanisms of cold signaling.

## Minty cool

The two populations of cold-sensitive cultured neurons described above can be furthered divided by their sensitivity to menthol, with the low-threshold cells being predominantly menthol-sensitive and the high-threshold population largely insensitive [15,17]. As capsaicin elicits a sensation of burning and tingling heat, cooling compounds, such as menthol, elicit the psychophysical sensation of cold [19]. Menthol, a cyclic terpene alcohol found in leaves of the genus *Mentha*, is used in a wide range of products, such as confectionary, candy, toothpastes, vapo-rubs, and aromatherapy inhalations. When applied at low concentrations to the skin or mouth, menthol elicits a pleasant cool sensation, while higher doses can cause burning, irritation, and pain [19-21]. In fact, menthol was recently shown to evoke pain in humans through activation and sensitization of C-fibers [22]. Conversely, prolonged exposure to large doses of menthol will adapt or desensitize cold-sensitive neurons, a process analogous to that of capsaicin and heat-sensitive fibers [20,23,24]. Menthol application in the mouth can transiently prevent the irritancy of concomitant or subsequent capsaicin exposure, but very few studies have directly assessed the analgesic properties of menthol and it remains to be seen if menthol or other cooling compounds can be used as effective analgesics [25].

While it had been known for centuries that menthol produces a sensation of cold, the molecular site of action was not known, nor was it known if menthol and cold activate sensory afferents through similar mechanisms. Over fifty years ago, Hensel and Zotterman, using lingual nerve recordings, proposed that menthol exerts its actions on cold-sensitive fibers by raising their temperature threshold and suggested that menthol specifically acts upon a cold receptor [26]. Recent support for this hypothesis came from a number of studies in cultured sensory neurons where ~10–20% of excitable cells were menthol-sensitive [13,14,27], similar to the numbers reported to be cold-sensitive. Additionally, menthol and cold were both shown to elicit non-selective cation currents in these cells (threshold temperature near 28°C) and menthol-induced responses were temperature dependent [13,14]. Therefore, it seemed likely that cold and menthol had a common molecular site of action, activating a Ca^2+ ^permeable channel.

## TRPM8, the minty-cool ion channel

The above hypothesis was confirmed by the concurrent cloning of a cold and menthol receptor by two independent groups; one using menthol to expression-clone a cDNA, from rat TG neurons, that could confer menthol-sensitivity in heterologous expression systems [14], and another that used a genomics approach to identify TRP channels expressed in mouse DRG neurons [28]. This cold and menthol receptor, termed CMR1 or TRPM8, was activated at a temperature threshold of ~28°C, with currents increasing in magnitude down to 8°C [14,28], thus spanning both innocuous cool to noxious cold temperatures. Additionally, the biophysical properties of cold- and menthol-induced currents (ion selectivity, rectification, menthol EC_50_, and temperature activation threshold) in heterologous cells expressing TRPM8 were reminiscent of those recorded in sensory neurons [13,14]. TRPM8 transcripts are expressed in <15% of small diameter sensory neurons, consistent with the proportion of excitable cells shown to be cold and menthol-sensitive [14,28]. While the size of TRPM8 expressing neurons suggests them to be C-fibers, these cells do not express other markers such as TRPV1, neurofilament, or calcitonin gene-related peptide (CGRP) [28]. Thus, TRPM8 is not expressed in a class of afferents historically considered to be nociceptors [29].

In addition to menthol, a number of cooling agents, including icilin, eucalyptol, and WS-3, activate TRPM8 *in vitro *[14,30]. The former of these compounds, icilin, is considered a super-cooling agent since it has higher potency and efficacy than menthol in cellular and behavioral studies [14,31]. However, icilin appears to activate the channel in a manner that is divergent from other agonists, including cold. It was first reported that icilin can only activate TRPM8 in the presence of extracellular calcium [14], and Chuang et al. have further described the dependence of icilin activation of TRPM8 on calcium [32]. Indeed, TRPM8 acted as a coincidence detector of icilin and calcium in that a rise in intracellular calcium, either through influx via TRPM8 or release from intracellular stores, was required for icilin-induced TRPM8 currents. This study also mapped a critical amino acid residue required for icilin activity to a single glutamine in the third transmembrane domain of the channel. Interestingly, work from the same laboratory had previously determined that the capsaicin-binding site in TRPV1 maps to the same region [33], suggesting a conserved mechanism for ligand activation of these thermosensitive channels [32]. However, it should be noted that this single residue in TRPM8 does not appear to be involved in the menthol- and cold-sensitivity of the channel, thus suggesting that the TRPM8 can be gated by distinct mechanisms.

Along with the number of cooling agents that activate TRPM8, several antagonists have been identified, including BCTC, thio-BCTC, capsazepine, and protons [30,34]. The latter of these findings has further supported the notion of differential modulation of TRPM8 by various mediators. Specifically, lowering of the intracellular pH to below 7 was able to completely block TRPM8 currents elicited by either cold or icilin, but not menthol [34]. However, Behrendt et al. reported that both menthol- and icilin-responsiveness were reduced by lowering external pH (cold was not tested) [30]. Interestingly, in the former study, changes in intracellular pH dramatically altered the thermal threshold for activation of the channel, suggesting that intracellular acidity has some regulatory role in this regard. However, it still remains to be seen if the pH-dependence of TRPM8 can be placed in a physiological context, such as inflammatory injury.

Cold receptors will adapt *in vivo *with prolonged cold stimulation [8,35], a phenomena also observed in recordings of cultured sensory neurons [13,36]. Menthol and cold-evoked currents in cells heterologously expressing TRPM8 will also adapt to prolonged stimuli in a manner that is dependent upon calcium [14], similar to capsaicin-induced desensitization of TRPV1 [37]. Interestingly, both menthol- and cold-induced adaptation were absent in recordings in excised patches from sensory neurons, suggesting this process is not an intrinsic property of the channel, but requires a cytoplasmic or membrane component lost upon membrane excision [24,36]. While the mechanism of adaptation is not well understood, recent findings by Liu and Qin [38] have suggested that TRPM8, like many TRPM channels [39,40], requires the presence of phosphatidylinositol 4,5-bisphosphate (PIP2) for activity. They demonstrated that menthol- and cold-evoked currents decreased or ran-down upon patch excision, a process that was inhibited under conditions of decreased phosphatase activity. Moreover, addition of exogenous PIP2 to the cytoplasmic face of the membrane recovered most of the menthol and cold-evoked currents. While the relationship between the effects of increased intracellular calcium in adaptation and PIP2-mediated channel rundown has not been established, these observations suggest that these two phenomena may be linked. Moreover, whether either is related to a mechanism of analgesia remains to be seen. Nonetheless, the cloning of TRPM8 established the first molecular detector of cold stimuli and its *in vitro *properties are consistent with this role *in vivo*. Furthermore, TRPM8 confirmed Hensel and Zotterman's half-century old hypothesis [26] and established that TRP channels can confer thermal stimuli over broad ranges of temperature.

## TRPA1, a noxious cold sensor?

While TRPV1 and TRPV2 established TRP channels as neuronal thermosensors, the cloning of TRPM8 suggested that detection of temperatures beyond the ranges of these channels may be conferred by other TRPs. Indeed, two members of the TRPV subfamily, TRPV3 and TRPV4, are involved in thermosensation of warmth [4]. In regard to cold sensation, as described above, a cold-sensitive, menthol-insensitive population of sensory neurons has been observed in culture, suggesting that these cells possess a cold thermosensor other than TRPM8 [15,17]. Story et al. first suggested that the TRP-like channel TRPA1 (or ANKTM1) mediates cold-responsiveness in these cells when they reported that noxious cold temperatures activated the mouse orthologue of this ion channel [41]. This channel was first identified as a transformation-sensitive RNA transcript in human fibroblasts [42]. However, TRPA1 transcripts were later found in a population of sensory neurons distinct from those expressing TRPM8, but almost exclusively in nociceptive afferents that also express TRPV1, Substance P, and CGRP [41]. Calcium microfluorimetry and voltage-clamp recordings performed using mTRPA1-expressing Chinese Hamster Ovary (CHO) cells demonstrated that temperatures, with an aggregate threshold of ~17°C (range between 8–28°C), elicited non-selective cation currents that were blocked by ruthenium red, a blocker of several Ca^2+^-permeable channels. Moreover, the cooling compound icilin, a known agonist for TRPM8 [14], also activated TRPA1 currents, although with reduced potency compared to TRPM8 [41]. Thus, due to its expression pattern and temperature threshold, TRPA1 was proposed to be a detector of noxious cold in nociceptive afferents [41].

However, the above findings were questioned when Jordt et al. reported the rat and human orthologues of TRPA1 to be receptors for isothiocyanates, the pungent ingredients in wasabi and yellow mustard, and proposed the channel mediates the inflammatory and vasodilatory effects of these agents [43]. The controversy arose when this study did not observe cold-activation of TRPA1 currents when the channel was heterologously expressed in either a human embryonic kidney (HEK293) cell-line or *Xenopus *oocytes. Similar results were recently reported by Nagata et al. (see below) [44]. Furthermore, currents elicited by allyl isothiocyanate, or mustard oil (MO), were reduced upon a reduction in temperature to beyond the thermal thresholds reported by Story et al. [41,43]. In addition to isothiocyanates, other pungent compounds were subsequently reported to activate TRPA1 *in vitro*, including the ingredients found in cinnamon (cinnamaldehyde), wintergreen, and clove oil, as well as ginger and methyl salicylate [45]. It should be noted that this latter report, from the same laboratory that originally reported cold-activation of TRPA1 [41], reproduced the earlier findings that the channel was sensitive to cold in both mammalian cells and *Xenopus *oocytes. In addition to these pungent compounds, the inflammatory peptide bradykinin also activated TRPA1 currents in a G-protein-coupled receptor-dependent manner, presumably via phospholipase C (PLC) [45]. Similarly, when TRPA1 was co-expressed with the PLC-coupled M1 muscarinic acetylcholine receptor (mACHR), application of acetylcholine elicited inward currents [43]. Thus, these data, and its expression in nociceptors, suggests that TRPA1 is involved in nociceptive signaling, and appears to mediate the distinct pungent sensations provided by a number of compounds. Moreover, it has also been postulated that TRPA1 plays an important role in inflammatory hypersensitivity in that the channel may be activated in a receptor-operated mechanism, perhaps through activation of PLC, by pro-algesic or pro-inflammatory mediators [43,45].

While the ability of TRPA1 to respond to temperature *in vitro *is still unresolved, a number of studies using cultured sensory neurons have further confused the issue. In the initial description of TRPA1, Story et al. reported that cold (average threshold temperature of ~15°C) and capsaicin activate a menthol-insensitive population of mouse DRG neurons in culture [41]. Thus, the pharmacology of these responses, as well as the high-threshold temperature for activation, suggested that TRPA1 accounts for the cold-sensitivity of these cells. This same group further supported these original findings, reporting that ~70% and 90% of cold-sensitive, menthol-insensitive mouse DRG neurons responded to MO and cinnamaldehyde, respectively [45]. However, a number of alternative studies have failed to reproduce these findings. First, Jordt et al. did not find evidence for a population of cultured rat TG neurons that were sensitive to both cold and MO, but not menthol [43]. Greater than 90% of the cold-sensitive neurons were menthol-sensitive, while those few cells that were cold- and MO-sensitive (~5%) also responded to menthol. Thus, the cold responses observed in this latter neuronal population were likely mediated by TRPM8. Secondly, Babes et al. recently suggested that TRPA1 does not mediate cold-responsiveness in cold-sensitive, menthol-insensitive neurons after they observed no correlation between cold sensitivity and MO responses in rat DRG cultures [17]. Lastly, two studies have provided indirect evidence supporting the notion that cold-sensitivity in high-threshold, menthol-insensitive cultured rat neurons is not mediated by TRPA1 [15,18]. These reports showed that the majority of these cells were labeled with the isolectin B4 (IB4), a marker for non-peptidergic sensory afferents [46]. Thus, since TRPA1 expression was shown to be exclusively in CGRP-positive mouse DRG cell bodies [41], this would preclude TRPA1 expression in IB4-positive nerves. However, there may be some significant differences in the phenotype of afferents in culture versus *in vivo*, due to growth factor-dependent [41], or independent mechanisms [17]. It should also be noted that species variations (mouse versus rat) may be attributing to these discrepancies.

The enigma of TRPA1 has been furthered by two additional findings. First, the *Drosophila melanogaster *orthologue of the channel was cloned and when expressed in heterologous expression systems, cold temperatures did not elicit membrane currents [47]. However, warm temperatures did activate dTRPA1 currents, within the range of 24–29°C, and flies with reduced expression of dTRPA1 exhibited deficits in normal thermotaxis to heat [48]. At the amino acid level, dTRPA1 is 32% identical and 54% similar to mTRPA1 [41]. In contrast, dTRPA1 is 22% identical and 39% similar to Painless, another drosophila TRP-like channel known to be involved in noxious thermal and mechanical signaling [49]. Thus, while dTRPA1 may be a relative sequence orthologue of the mammalian channels, it is not a functional one, and may be more related to Painless. Secondly, an alternative role for TRPA1 in sensory transduction has been proposed by Corey et al. and Nagata et al., reporting it as a candidate for the elusive mechanosensitive transduction channel in vertebrate hair cells [44,50]. These findings were based upon localization of mTRPA1 transcripts and protein in hair cells, deficits in hair cell function after inhibition of TRPA1 protein expression, and similar biophysical and pharmacological properties of heterologously expressed TRPA1 and the hair cell transduction channel. Moreover, in the latter study cold could not elicit currents in cells heterologously expressing mTRPA1 [44], similar to the findings of Jordt et al. for the rat and human orthologues of the channel [43]. Thus, while cold activation of TRPA1 remains puzzling, the channel may have a diverse range of biological roles that depends upon the species and the cellular context in which the channel is expressed.

## Conclusion

**Figure 1:**
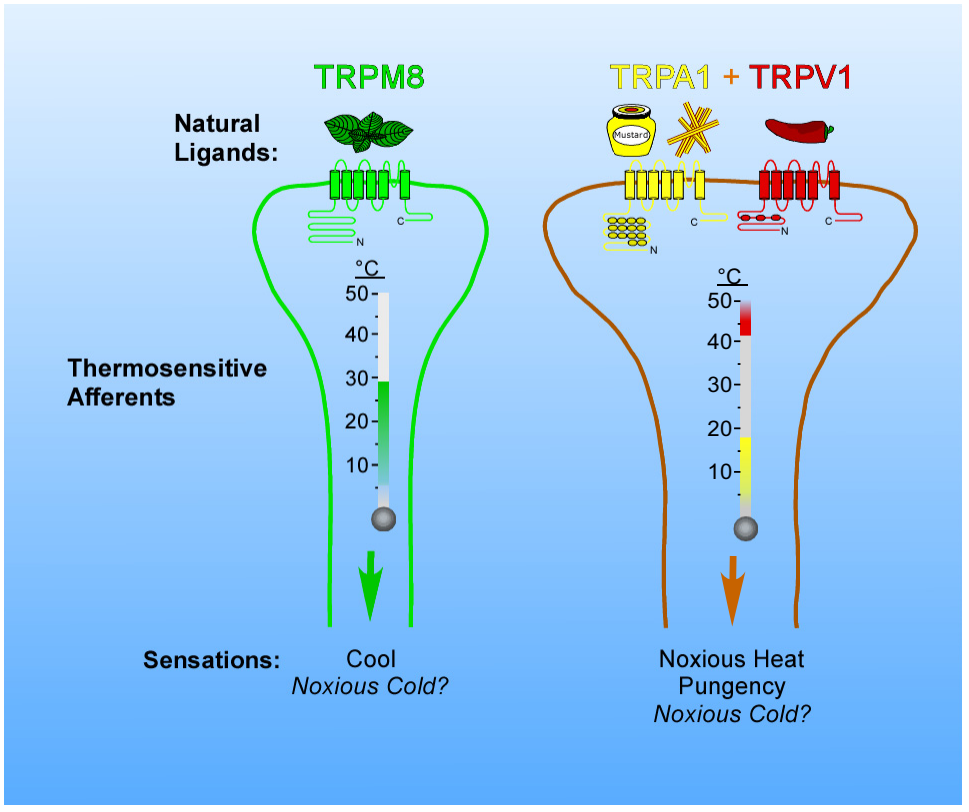
**Molecular identity of cold-sensitive afferents based upon the expression of TRPM8 and TRPA1**. TRPM8 and TRPA1 are found in distinct and non-overlapping populations of sensory afferents, with TRPA1 expressed exclusively in some, but not all, neurons that express the heat-gated channel TRPV1. These thermosensitive TRP channels respond to a number of naturally occurring pungent compounds, such as menthol (mint), allyl isothiocyanate (mustard oil), cinnamaldehyde (cinnamon), and capsaicin ('hot' chili peppers), thus providing a molecular explanation for how these compounds provide distinct sensations of cold, heat, or spiciness. Based upon *in vitro *characterizations of these channels, along with their distinct expression patterns, thermal stimuli activating TRPM8-expressing afferents elicit the sensation of cool to potentially noxious cold, while TRPA1 afferents will merge both noxious cold and noxious heat, due to the expression of TRPV1.

The past few years have firmly established mammalian TRP channels as the primary detectors of thermal stimuli in the peripheral nervous system. These channels can account for temperature perception over the entire perceived temperature spectrum and also play fundamentally important roles in nociceptive signaling [4]. In comparison to the wealth of published data on heat- and capsaicin-sensitive nerves, and TRPV1, our knowledge of cold sensation and the involvement of TRPM8 and TRPA1 are still in their infancy. Thus, several key and fundamental issues regarding cold sensation and these channels remains (Figure 1). First, while the sensitivity range of TRPM8 encompasses both innocuous and noxious temperatures, the role of the channel in nociception is still unknown. The fact that high-doses of menthol can produce pain would suggest that TRPM8 is nociceptively-relevant. However, it is uncertain if TRPM8 is the only menthol receptor in sensory afferents, or if menthol affects other biological processes in a nociceptively-relevant manner [19]. Second, it is not known if TRPM8 or TRPA1 will provide good targets for as yet unidentified analgesics that may alleviate chronically painful conditions such as cold allodynia. Naturally occurring products, such as menthol, mustard oil, and cinnamon, have been used for centuries in nociceptively-relevant manners. Thus, now that molecular targets for these and related compounds have been identified, critical approaches can be developed to determine the role of these channels in nociception, and if compounds that modulate them can be used therapeutically. Lastly, whether or not TRPA1 is involved in cold-sensation needs to be reconciled by genetic approaches, such as TRPA1-null mice, or neuronal membrane current recordings combined with antibody labeling, studies undoubtedly currently underway in a number of laboratories. Until these and other, more directed approaches are performed, the evidence for or against TRPA1 functioning as a cold-sensor *in vivo *is conflicting. Therefore, both TRPM8 and TRPA1 are and will remain fascinating molecules to study and, even though there is considerable debate over these thermosensors, as written by the 19^th ^century essayist Lyman Beecher *"No great advance has ever been made in science, politics, or religion, without controversy." *[51].
